# Supplementary material for: An approach towards a perfect thermal diffuser
Source: Sci Rep. 2016 Jul 11;6:29649. doi: 10.1038/srep29649 (PMC4939596; doi:10.1038/srep29649)
Supplement: Supplementary Information [file srep29649-s1.pdf]

<u>Supplementary Information</u>

An approach towards a perfect thermal diffuser

Krishna P. Vemuri and Prabhakar R. Bandaru,

Department of Mechanical Engineering, University of California, San Diego,

La Jolla, CA 92093-0411

I. Obtaining a uniform temperature distribution (rectangular space) and isotropic heat flow (spherical space), through mutual mapping

The mapping transformation involves the mapping(*1*) of a rectangular block, formed from an isotropic material of thermal conductivity: κ, and of length: L Figure S1 (a), onto a cylindrical sector: Figure S1 (b), The correspondent mapping between a space spanned in rectangular geometry, *i.e.,* **U** = (x, y, z) to that of a space spanned by cylindrical geometry*, i.e.,* **V** = (X, Y, Z), accomplishes the incorporation of features relevant to a uniform temperature distribution coupled with isotropic diffusion.

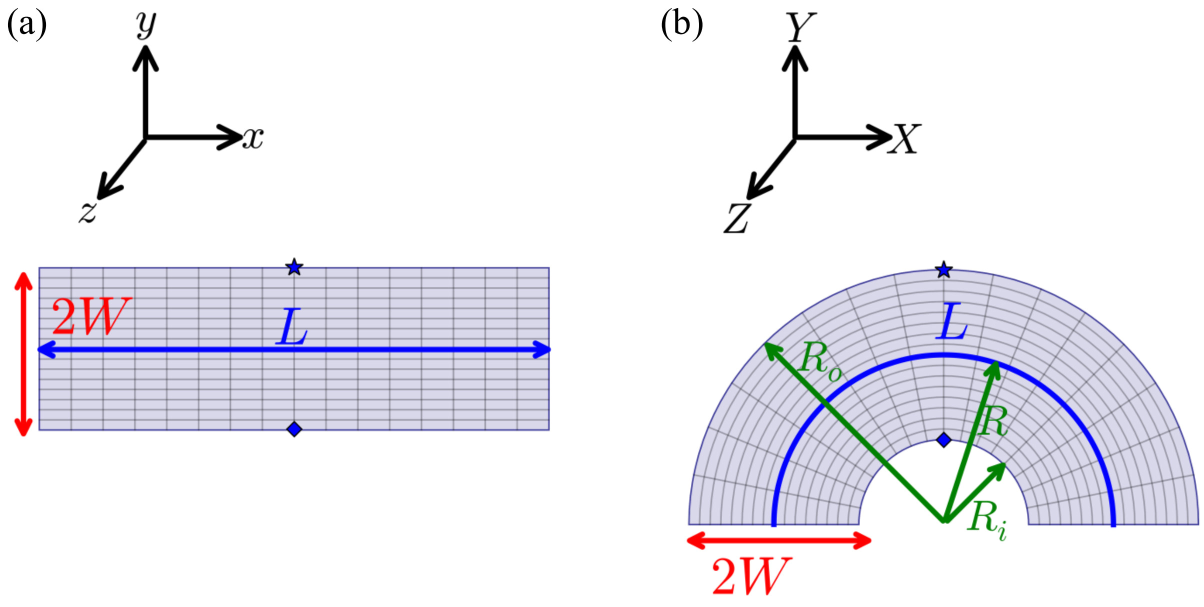

Figure S1 Schematic mapping of a **(a)** rectangular block of length L, width $2W$ and thickness t mapped onto **(b)** a cylindrical sector with central angle 2θ, inner radius L/θ-W, outer radius $L/\theta+W$ and thickness t. Two representative correspondent points (indicated, for example, by the ◆ and ★) are indicated. Note that, as depicted, $\theta=\pi/2$, $R_i=L/\theta-W$, $R_o = L/\theta+W$, R_o - R_i = $2W$.

The mapping of the (*x, y, z*) space to (X, Y, Z) space, indicated in Eqn. S1, below, is conformal and preserves the parallelism of the isotherms in the two spaces. The mutual geometric relationships are represented through:

$$X = ySin\left(\frac{x}{R}\right); Y = yCos\left(\frac{x}{R}\right); Z = z \qquad \text{(S1a)}$$

or, equivalently

$$x = R\,\mathrm{Tan}^{-1}\left(\frac{X}{Y}\right),\ y = \sqrt{X^2 + Y^2}\ , z = Z \qquad \text{(S1b)}$$

The Jacobian (*J*), for the transformation, is given by(*2–4*) $J_{U \to V} = \dfrac{\partial(X,Y,Z)}{\partial(x,y,z)}$:

$$J_{U \to V} = \begin{pmatrix} \frac{y}{R}\mathrm{Cos}\left(\frac{x}{R}\right) & \mathrm{Sin}\left(\frac{x}{R}\right) & 0 \\ \frac{-y}{R}\mathrm{Sin}\left(\frac{x}{R}\right) & \mathrm{Cos}\left(\frac{x}{R}\right) & 0 \\ 0 & 0 & 1 \end{pmatrix} \qquad \text{(S2)}$$

An alternate representation related to **Eqn. (S2)** involves the use of a cylindrical coordinate system (from Eqn. S1b), and with reference to the rectangular coordinate system at the top of Figure S1(b), and uses:

$$\begin{aligned} x &= R\,\mathrm{Tan}^{-1}(\frac{X}{Y}) = R\theta \\ y &= \sqrt{X^2 + Y^2} = r \\ z &= Z \end{aligned} \qquad \text{(S3)}$$

The modification of the thermal conductivity, due to the transformation given by Eqn. (S1), is then obtained through:

$$\kappa^{rect}\left(r,\theta,Z\right)=\frac{J_{U\to V}\kappa J_{U\to V}{}^{T}}{Det(J_{U\to V})}=\begin{pmatrix} \frac{r}{R}\mathrm{Cos}^2\left(\theta\right)+\frac{R}{r}\mathrm{Sin}^2\left(\theta\right) & \frac{1}{2Rr}\left(R^2-r^2\right)\mathrm{Sin}\left(2\theta\right) & 0 \\ \frac{1}{2Rr}\left(R^2-r^2\right)\mathrm{Sin}\left(2\theta\right) & \frac{r}{R}\mathrm{Sin}^2\left(\theta\right)+\frac{R}{r}\mathrm{Cos}^2\left(\theta\right) & 0 \\ 0 & 0 & \frac{R}{r} \end{pmatrix}\kappa \quad \text{(S4)}$$

The equivalent thermal conductivity in a rectangular (*X, Y, Z*) coordinate system is done through a rotation by an angle θ about the *Z*-axis, as follows:

$$\kappa^{circ}(X,Y,Z)=\frac{J_2\kappa^c(r,\theta,Z)J_2{}^{T}}{Det(J_2)}=\begin{pmatrix} \kappa_r^c & 0 & 0 \\ 0 & \kappa_\theta^c & 0 \\ 0 & 0 & \kappa_z^c \end{pmatrix}=\begin{pmatrix} \frac{R}{r} & 0 & 0 \\ 0 & \frac{r}{R} & 0 \\ 0 & 0 & \frac{R}{r} \end{pmatrix}\kappa \quad \text{(S5)}$$

where, $J_2=\begin{pmatrix} Cos\theta & -Sin\theta & 0 \\ Sin\theta & Cos\theta & 0 \\ 0 & 0 & 1 \end{pmatrix}$

Such a form has been indicated as Eqn (2) of the main text and the schematic variation of conductivity (κ_r^c ,κ_z^c , and κ_θ^c , has been plotted in **Fig. 3(A)**, in the main text.

II. Constructing the spatial anisotropy represented in Eqn. (2)/(S5) using isotropic materials

The spatial anisotropy represented through the relation in Eqn. (2) of the main text or (S5) in Section I of the Supplementary information, can be effectively reduced to a two-dimensional form, in the (r, θ) system, as $\kappa_r^c = \kappa_Z^c = \frac{R}{r}$. Also, it is noted from (S5) and Figure 3(a), that

$$\begin{aligned} \kappa_r^c &> \kappa_\theta^c \ ; \ R_i \le r < R \\ \kappa_r^c &= \kappa_\theta^c \ ; \ r = R \\ \kappa_r^c &< \kappa_\theta^c \ ; \ R < r \le R_o \end{aligned} \qquad \text{(S6)}$$

A composite thermal conductivity satisfying the above conditions can be obtained through the arrangement of two layers, with isotropic thermal conductivity values of κ_1 and κ_2. In our previous work(*2*, *7*), it was shown that the effective thermal conductivity of such layers arranged in parallel, *i.e.,* κ_p was larger than that of layers arranged in series, parallel, *i.e.,* κ_s.

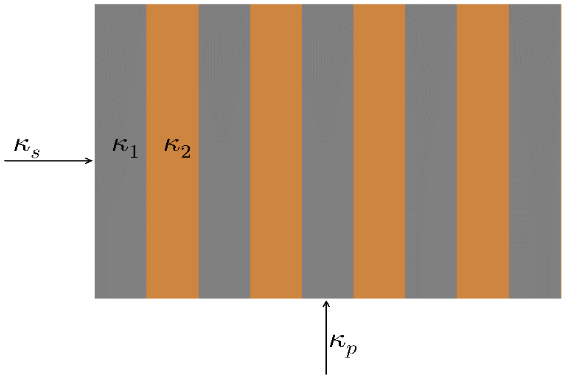

Figure S2 The alternate stacking of two layers, with isotropic thermal conductivity values of κ_1 and κ_2, respectively, can yield an effective composite thermal conductivity measured in series, *i.e.,* κ_s, and another *larger* value for parallel measurement, *i.e.,* κ_p.

For example, with reference to Figure S2, it may be derived that:

$$\kappa_p = \frac{\kappa_1 + \kappa_2}{2} \ , \ \kappa_s = \frac{2\kappa_1\kappa_2}{\kappa_1 + \kappa_2} \qquad \text{(S7a)}$$

Then,

$$\kappa_1 = \kappa_p + \sqrt{\kappa_p^2 - \kappa_p\kappa_s} \ , \ \kappa_2 = \kappa_p - \sqrt{\kappa_p^2 - \kappa_p\kappa_s} \qquad \text{(S7b)}$$

Consequently, for $r < R$, the orientation of layered elements would need to be parallel to the heat flux, with a correspondence of the κ_r^c ($/\kappa_z^c$) to κ_p and with κ_θ^c being related to the κ_s. However, for $r > R$, the orientation of layered elements would need to be arranged *in series* with the heat flux, with a correspondence of the κ_r^c ($/\kappa_z^c$) to κ_s and κ_θ^c related to κ_p. This would then imply a spatially varying κ_1 (r) and κ_2 (r).

III. Temperature variation in thermal diffuser employing spatial conductivity modulation

We consider cylindrical diffusers, of the type shown in Fig. 2(A) – in the main text, or Figure S1 (b), with an input heat flux q_{in} at r = R_i (= R-W) placed in an ambient (at a temperature = T_{amb}). A given convective heat transfer coefficient h between the outer surface at r = R_o (= R+W) and the ambient is assumed (*5*, *6*). The resulting temperature, at the outer surface, for the cylindrical diffusers, with (a) homogeneous and isotropic material ($T_{R_O}^{iso}$), and (b) with anisotropic and spatial modulation of the constituents ($T_{R_O}^{aniso}$), are then derived.

From thermal energy conservation,

$$q_{in}R_i t = hR_o t\left(T_{R_o}^{iso} - T_{amb}\right) = hR_o t\left(T_{R_o}^{aniso} - T_{amb}\right)$$

$$T_{R_O}^{iso} = T_{R_O}^{aniso} = T_\infty + \frac{q_{in}}{h}\left(\frac{R_i}{R_o}\right) \tag{S8}$$

(a) *Temperature* T_r^{iso} *in an isotropic cylindrical diffuser*

From heat flux continuity, for an isotropic cylindrical diffuser κ,

$$q_{in}R_i t = -\kappa r t\frac{\partial T(r)}{\partial r} \tag{S9}$$

Integrating Eqn. (S7) between r =r (at a temperature, T = T_r^{iso}) and r = R_o (at T = $T_{R_O}^{iso}$) and using the relation in (S6) for the boundary conditions, we get,

$$T_r^{iso} = \frac{q_{in}R_i}{\kappa}\ln\left(\frac{R_o}{r}\right) + T_{amb} + \frac{q_{in}}{h}\left(\frac{R_i}{R_o}\right) \tag{S10}$$

Consequently,

$$T_{r=R_i}^{iso} = q_{in}\left[\frac{R_i}{\kappa}\ln\left(\frac{R_o}{R_i}\right) + \frac{1}{h}\left(\frac{R_i}{R_o}\right)\right] + T_{amb} \tag{S11}$$

This is **Eqn. (3a)** in the main text.

(b) *Temperature* T_r^{aniso} *in an anisotropic cylindrical diffuser*

From heat flux continuity, for an anisotropic cylindrical diffuser, with spatially varying thermal conductivity, given by $\kappa^c(r,\theta,Z) = \kappa_r^c = \frac{R}{r}\kappa$, the heat conservation equation yields:

$$q_{in} R_i t = -\kappa_r^c r t \frac{\partial T(r)}{\partial r} \tag{S12}$$

Integrating Eqn. (S12) between $r = r$ (at $T = T_r^{aniso}$) and $r = R_o$ (at $T = T_{R_O}^{aniso}$) and using the boundary conditions represented in (S8), we get,

$$T_r^{aniso} = \frac{q_{in} R_i}{\kappa R}(R_o - r) + T_{amb} + \frac{q_{in}}{h}\left(\frac{R_i}{R_o}\right) \tag{S13}$$

Then,

$$T_{r=R_i}^{aniso} = q_{in}\left[\frac{R_i}{\kappa R}(R_o - R_i) + \frac{1}{h}\left(\frac{R_i}{R_o}\right)\right] + T_{amb} \tag{S14}$$

This is **Eqn. (3b)** in the main text.

IV. The arrangement of materials used for simulation in Figure 5

We indicate a representative material arrangement underlying the computational simulations needed to validate our analytical derivations and the principles underlying the perfect thermal diffuser design. Following Fig. 2(B), we use representative R_i = 0.5 cm, R_o = 4.5 cm (implying R = 2.5 cm and W = 2 cm), and L = 2.5 π cm, with $\kappa = 60\,\mathrm{W/mK}$.

From Eqn. (2)/ Eqn. S5, we obtain:

$$\kappa^{circ}(X,Y,Z) = \begin{pmatrix} \kappa_r^c & 0 & 0 \\ 0 & \kappa_\theta^c & 0 \\ 0 & 0 & \kappa_z^c \end{pmatrix} = \begin{pmatrix} \frac{R}{r} & 0 & 0 \\ 0 & \frac{r}{R} & 0 \\ 0 & 0 & \frac{R}{r} \end{pmatrix} \kappa = \begin{pmatrix} \frac{150}{r} & 0 & 0 \\ 0 & 24r & 0 \\ 0 & 0 & \frac{150}{r} \end{pmatrix} \quad \text{(S15)}$$

At any given r, the thermal conductivity mimicking the spatial anisotropy may be determined using the above relation. We approximately sub-divide the diffuser into four regions of radial thickness 1 cm. The κ_r^c $(/\kappa_z^c)$ and κ_θ^c is subsequently computed from (S15), at any given r (taken for example, to be at the center of the individual regions, *e.g.,* for the first region spanning r = 0.5 cm and r = 1.5 cm, the *effective* r = 1 cm. Following the rationale presented in Section III of the Supplementary Materials, for $r < R$, the κ_r^c $(/\kappa_z^c)$ is corresponded to a κ_p and the κ_θ^c is related to the κ_s. Consequently, the appropriate κ_1 and κ_2 is obtained through using Eqn. (S7b). Such details for all the four sections of the anisotropic diffuser are depicted in Table I, on the next page.

For a point ***at the maximum*** of the curve in **Figure 4**, *i.e.,* at R = 2.5 cm and W = 2 cm

Table S1 The corresponding arrangement of materials in Fig. 5(A). It is discussed in the paper as to how the arrangement of isotropic materials in an anisotropic manner could yield uniform temperature distribution along with a lower source temperature. Following Fig. 2(B), R_i = 0.5 cm, R_o = 4.5 cm, and L = 2.5 π cm, with a representative κ=60 W/mK. The 4 cm (= $R_o - R_i$) wide strip is divided into four 1 cm wide **regions** (Column 1), with an average ***r*** (Column 2), and the use of Eqn. (S15) to compute the κ_r^c (Column 3) and κ_θ^c (Column 4). Consequently, the relations in Eqn. S7b, were used to obtain κ_1 (Column 5) and κ_2 (Column 6).

Region	Average "*r*"	κ_r (W/mK)	κ_θ (W/mK)	κ_1 (W/mK)	κ_2 (W/mK)
0.5 – 1.5 cm	1 cm	148	24	282 (= ***a***)	12 (= ***b***)
1.5 – 2.5 cm	2 cm	74	47	118 (= ***c***)	29 (= ***d***)
2.5 – 3.5 cm	3 cm	49	71	110 (= ***e***)	31 (= ***f***)
3.5 – 4.5 cm	4 cm	37	94	169 (= ***g***)	20 (= ***h***)

For a point ***away*** from the maximum, *i.e.,* at R = 5 cm and W = 2 cm

For a diffuser needing to dissipate a q_{in}, of 2x10^6 W/m^2, the corresponding anisotropic thermal conductivity was derived assuming a nominal isotropic κ = 60W/mK and T_{amb} = 293K. A comparison of the analytically predicted temperature variation with the computational simulation results is illustrated in **Figure S3 (b),** and found to be linear, in excellent accord with predictions (Figure 4). A reduction in the source temperature by ~ 50 K in the engineered composite was observed, corresponding to temperatures at the center (r= R_i) of 1200 K and 1248 K, for the anisotropic and the isotropic cases, respectively.

(A) **(B)**

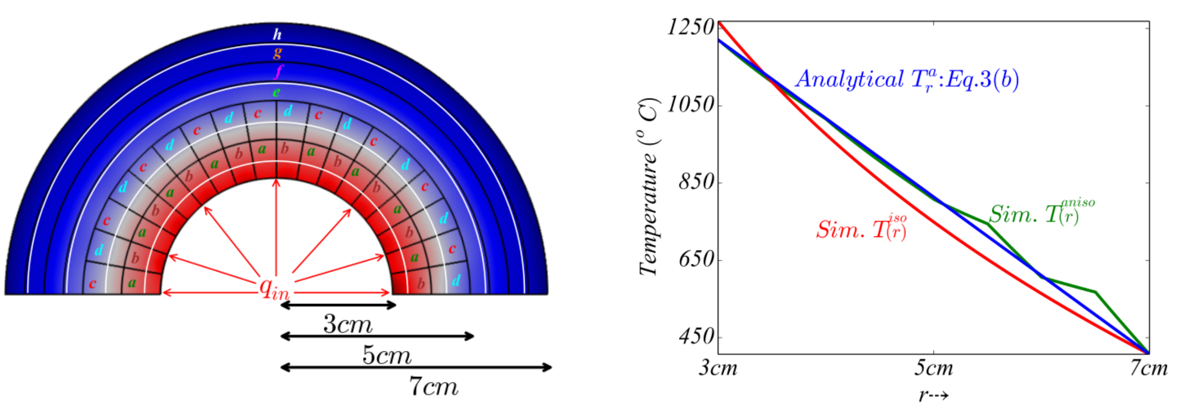

Figure S3 (A) A uniform temperature distribution in the composite was obtained through the use of an anisotropic arrangement, composed of materials of constant and isotropic thermal conductivity. The symbols refer to the thermal conductivity of the isotropic materials, and are indicated in **Table S2** below. **(B)** The resulting temperature variation, from the simulations, was in excellent accord with the analytical relation derived in Eqn. 3(b).

Table S2 The corresponding arrangement of materials in Fig. S3(a).

Region	Average "*r*"	κ_r (W/mK)	κ_θ (W/mK)	κ_1 (W/mK)	κ_2 (W/mK)
3 – 4 cm	3.5 cm	84	42	145 (= ***a***)	24 (= ***b***)
4 – 5 cm	4.5 cm	66	53	94 (= ***c***)	37 (= ***d***)
5 - 6 cm	5.5 cm	54	65	92 (= ***e***)	38 (= ***f***)
6 – 7 cm	6.5 cm	45	77	126 (= ***g***)	28 (= ***h***)
